# Scrotal Swelling and Testicular Atrophy due to Schistosomiasis in a 9-Year-Old Boy: A Case Report

**DOI:** 10.1155/2011/787961

**Published:** 2011-06-30

**Authors:** Peter F. Rambau, Alphonce Chandika, Philipo L. Chalya, Kahima Jackson

**Affiliations:** ^1^Department of Pathology, Weill Bugando University College of Health Sciences, P.O. Box 1464, Mwanza, Tanzania; ^2^Department of Surgery, Weill Bugando University College of Health Sciences, P.O. Box 1464, Mwanza, Tanzania; ^3^Department of Pathology, Bugando Medical Center, P.O. Box 1370, Mwanza, Tanzania

## Abstract

Schistosomiasis is a communicable disease which commonly involves urinary bladder causing hematuria, or large bowel causing bloody stool. The common species encountered in this lake region surrounding Lake Victoria in Tanzania are *Schistosoma haematobium* and *Schistosoma mansoni*. Complications can lead to portal hypertension due portal fibrosis in liver, and fibrosis in lung can lead to pulmonary hypertension; this commonly seen with *S. mansoni*. Major complications of *S. maeametobium* are chronic cystitis with squamous metaplasia with subsequent development of squamous cell carcinoma. Involvement of spinal cord causing paraplegia has been observed in *S. haematobium*. Other unusual pathology of schistosomiasis has been described, such as involvement of the appendix, ovary, prostate, and cervix. Here, we present a case of schistosomiasis in a 9-year-old boy who presented with left scrotal pain for one year which was accompanied by scrotal swelling; surgical exploration was done, and the finding was hydrocele and atrophic testes with nodules on the surface. Histological examination reveals atrophic testis and heavy active granulomatous inflammation with schistosoma eggs consistent with *Schistosoma haematobium* in the tunica vaginalis.

## 1. Introduction

Schistosomiasis is endemic disease in Tanzania, and it is one of the most important causes of morbidity with significant mortality. In Tanzania, the common encountered species are *S. mansoni* and *S. haematobium*, which causes intestinal schistosomiasis and urinary schistosomiasis, respectively. The common symptoms are blood in stool and hematuria. Significant morbidity is seen in those patients who develop complications such as end-stage renal diseases, chronic liver diseases with portal hypertension, and cancers associated with schistosomiasis [1–3]. Unusual presentations of schistosomiasis have been described in many organs in the body, and commonly, these lesions are not clinically suspicious for schistosomiasis, and most of them are diagnosed histologically as incidental findings. In our settings, such lesions have been described in appendix causing perforation with peritonitis [4], and schistosomal eggs was also seen within the prostate cancers [5]. Ovarian schistosomiasis has been described causing chronic granulomatous inflammation producing ovarian pseudotumor, and it was also seen in fallopian tube in association with carcinoma [6, 7]. Schistosomiasis of the cervix presents like other cervical condition such as cervicitis or cancer, and cervical cancer with schistosoma eggs had been described in Tanzania [8]. 

Testicular schistosomiasis is not commonly reported, and some cases have been reported due to *S. mansoni*. Testicular schistosomiasis can present as testicular nodule [9], and sometimes, it can mimic carcinoma leading to unnecessary orchidectomy [10, 11]. In epididimis, schistosomal causes scrotal pain, and involvement of seminal vesicles has been documented [12, 13]. Schistosomiasis of the scrotum has also been described, causing hydrocele or causing chronic dermatitis [14, 15]. Here, we presents a case of a 9-year-old boy presented with left scrotal swelling and pain for one year which was later diagnosed histologically to be schistosomiasis after exploration and orchidectomy.

## 2. Case Report

A 9-year-old boy was referred from Magu district hospital to Bugando Medical center with history of left scrotal pain and swelling for one year. The swelling was progressive increasing in size, and this was accompanied by pain, and he had no history of fever, weight loss, or evening fevers. There was no history of hematuria in the past or during the course of his illness. The patient had been using local medicine of unknown nature without improvement. At district hospital, antibiotics and analgesic drugs were given without improvement, and the patient was referred to Bugando Medical Center. On examination, the boy was well nourished with no jaundice or pallor. All the system was essentially normal. Local examination revealed left scrotal swelling, slightly tender, cystic in consistence, could not go above it, and it was irreducible, and the impression of hydrocele was made. The patient was planned for hydrocelectomy. Initial investigation showed hemoglobin of 11.5 gm/dL, blood group B Rh+, and urine and stool was normal with no ova. The patient underwent surgery; the findings were a hydrocele, thickened tunica and small nodules on the surface of the testis which created suspicion for testicular tumor, and orchidectomy was done. The patient had uneventful recovery and was discharged after seven days.

**Figure 1:**
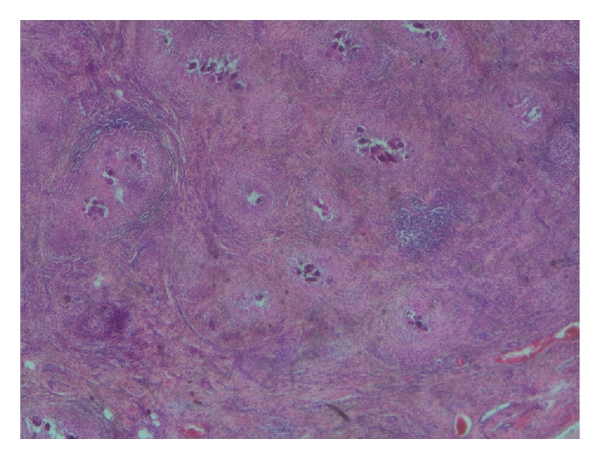
*H and E* stain showing chronic granulomatous inflammation in tunica with schistosoma eggs (×2).

**Figure 2:**
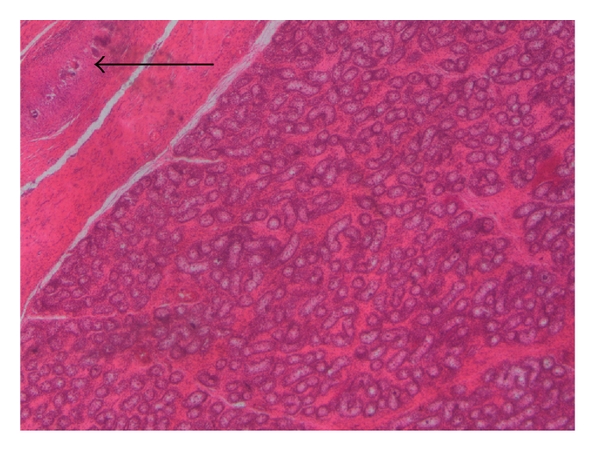
*H and E* stain showing atrophic testis and granulomatous lesion and schistosoma eggs in tunica  (see the arrow) (×2).

**Figure 3:**
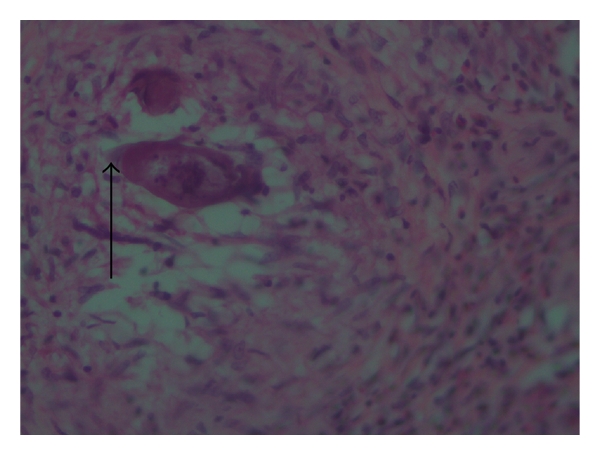
*H and E* stain showing typical *S. hematobium* egg with terminal spine (see the arrow) (×20).

At histopathology department, a cystic wall of 4 × 4 × 3 cm was received, and a testis of 1 × 0.5 cm, looked atrophic and yellowish with firm nodules on its surface. Histological examination with haematoxylin and eosin stain (*H and E*) showed active chronic granulomatous inflammation with epithelioid macrophages and foreign body giant cells and numerous schistosomal eggs in the thickened tunica albugenia with eosinophils infiltrate. The testis was not involved, and it showed atrophic changes with fibrosis; epididymis was also normal showing only dilatation. Close examination of the eggs showed the terminal spine consistent with *S. haematobium*; the eggs was not calcified (see Figures 1, 2, and 3), indicating active inflammation. Followup of the patient in outpatient there was no further complaints, and the patient was given Praziquantel 400 mg start.

## 3. Discussion

Presentation of scrotal swelling and pain clinically will not raise suspicion for schistosomiasis as seen in this patient; findings of hydrocele should rise the suspicious for filariasis, which is also not so common in this region. The finding of thickened tunica vaginalis together with nodules on the testis may also raise the suspicion for testicular tumors which are common at this age. Tuberculous orchitis commonly tends to be misdiagnosed as a tumor as well, and tuberculosis is very common in our setting. In this endemic area for schistosomiasis, the common mode of presentation is hematuria and bloody stool; scrotal swelling is very exceptional, and this is why clinically it was not part of differential diagnosis. Absence of common symptoms of schistosomiasis and the finding of no ova in the urine or stool brought the challenge for diagnosis of schistosomiasis in this patient. 

Pathogenesis of schistosomal infection involving the testis is through the larva migration from the lungs to the veins, where the adult lodge into genitourinary venous plexus, and the excretion of the eggs causes chronic granulomatous inflammation. For our patient, there was no urinary bladder symptoms, and the patient had no history of hematuria in the past; there is possibility of the adult worms to settle in scrotal and testicular venous plexus only, or there was subclinical infection in the urinary bladder, and a subclinical painless testicular lesion due to schistosomiasis has been reported in the past [16, 17]. Findings of hydrocele can be explained by chronic granulomatous inflammation causing obstruction of lymphatics in tunica and extravasation of inflammatory fluid exudates due to schistosomal eggs, as the eggs were still viable (not calcified) with active inflammation. Testicular atrophy can be explained by chronic inflammation and fibrosis with tension in the tunica due to inflammatory exudates causing compression of the testis and minor ischemia. 

Noninvasive techniques like ultrasound can detect schistosomiasis as hypoechoic lesion, which should raise the suspicion for granulomatous process, and MRI (Magnetic resonant image) is very sensitive by showing irregular tunica [9]. When the lesion is located within the testis, commonly it shows heterogeneous echotexture similar to that of most testicular cancers, and shistosomiasis involving the testis should be one of the differential diagnosis in testicular cancers; however, other numerous scrotal infections such as filariasis and paracoccidioidomycosis can produce the same picture, and this create more diagnostic challenges [18, 19]. In this patient, sonography was not done, as this could be of more use and MRI is not available in our center. Use of frozen biopsy in suspicious testicular lesion has been useful in diagnosis, and this avoid unnecessary orchidectomy [16]; however, in our setting, frozen section is not available. Clinical suspicion and use of ultrasound in our setting should be applied to scrotal swelling to avoid unnecessary orchidectomy.

## 4. Conclusion

Although schistosoma associated scrotal conditions are rare, surgeons should bear it on mind when dealing with scrotal swellings especially in this region, where schistosomiasis is endemic. Routine ultrasound investigation for scrotal swelling should be emphasized, as unnecessary orchidectomy will be avoided.

##  Conflict of Interests

The authors declare that there is no conflict of interests in this paper.

##  Consent

The father of the boy consented for the publication of these findings.
